# A method for predicting postpartum depression via an ensemble neural network model

**DOI:** 10.3389/fpubh.2025.1571522

**Published:** 2025-04-14

**Authors:** Yangyang Lin, Dongqin Zhou

**Affiliations:** ^1^School of Smart Health Care, Zhejiang Dongfang Polytechnic, Wenzhou, China; ^2^Nursing Teaching and Research Department, Wenzhou People's Hospital, Wenzhou, China

**Keywords:** postpartum depression, neural networks, machine learning, clinical decision-making, postpartum women

## Abstract

**Introduction:**

Postpartum depression (PPD) has numerous adverse impacts on the families of new mothers and society at large. Early identification and intervention are of great significance. Although there are many existing machine learning classifiers for PPD prediction, the requirements for high accuracy and the interpretability of models present new challenges.

**Methods:**

This paper designs an ensemble neural network model for predicting PPD, which combines a Fully Connected Neural Network (FCNN) and a Neural Network with Dropout mechanism (DNN). The weights of FCNN and DNN in the proposed model are determined by their accuracies on the training set and respective Dropout values. The structure of the FCNN is simple and straightforward. The connection pattern among the neurons of the FCNN makes it easy to understand the relationship between the features and the target feature, endowing the proposed model with interpretability. Moreover, the proposed model does not directly rely on the Dropout mechanism to prevent overfitting. Its structure is more stable than that of the DNN, which weakens the negative impact of the Dropout mechanism on the interpretability of the proposed model. At the same time, the Dropout mechanism of the DNN reduces the overfitting risk of the proposed model and enhances its generalization ability, enabling the proposed model to better adapt to different clinical data.

**Results:**

The proposed model achieved the following performance metrics on the PPD dataset: accuracy of 0.933, precision of 0.958, recall of 0.939, F1-score of 0.948, Matthews Correlation Coefficient (MCC) of 0.855, specificity of 0.923, Negative Predictive Value (NPV) of 0.889, False Positive Rate (FPR) of 0.077, and False Negative Rate (FNR) of 0.061. Compared with 10 classic machine learning classifiers, under different dataset split ratios, the proposed model outperforms in terms of indicators such as accuracy, precision, recall, and F1-score, and also has high stability.

**Discussion:**

The research results show that the proposed model effectively improves the prediction performance of PPD, which can provide guiding suggestions for relevant medical staff and postpartum women in clinical decision-making. In the future, plans include collecting more disease datasets, using the proposed model to predict these diseases, and constructing an online disease prediction platform to embed the proposed model, which will help with real-time disease prediction.

## 1 Introduction

Postpartum depression, also known as puerperal depression, is an emotional disorder caused by childbirth (1). Medically, PPD is referred to as severe depression with postpartum onset (2, 3), generally considered to be a depressive episode occurring within 4 weeks after childbirth (4, 5). However, psychological and social factors can significantly affect the onset of PPD (6). For example, the occurrence of major unexpected events in life or the accumulation of daily trivialities can make new mothers burdened excessively (7). The lack of social support, such as insufficient support from partners, family, and friends, can cause new mothers to feel isolated and uneasy. The psychological factors of new mothers, such as specific personality traits, past mental health problems, and negative cognitive styles, can increase the risk of developing PPD. The role pressure brought about by the transition of new mothers also makes PPD more likely to occur (8, 9). The symptoms of PPD usually include sleep disturbances, anxiety, irritability, and feeling overwhelmed, excessive concern about the baby’s health and feeding, suicidal thoughts, and worries about harming the baby (10).

PPD can even drive mothers to have suicidal thoughts, which severely impacts their mental health (11, 12). PPD can trigger negative physical symptoms in mothers, such as insomnia, loss of appetite, and body aches, which can affect their daily lives and physical health (13, 14). Research has consistently shown that PPD has a negative impact on parent-infant interactions and infants’ cognitive, social, and emotional development (15). Because PPD can lead to difficulties in breastfeeding and reduced parent–child interaction, it prevents mothers from effectively caring for and attending to their newborns. Intervention efforts should focus on identification and treatment of PPD as early as possible to mitigate detrimental long-term impacts on parent–child relationships (16, 17). This situation not only affects the healthy growth of the baby but also exacerbates the mother’s feelings of guilt (18). Mothers with PPD tend to avoid social contact, resulting in a smaller social circle and an increased psychological burden (19). Mothers with PPD provide relatively less nutrition and care for their newborns, leading to restricted growth and development of the infants (20). The mother’s depressive emotions can have a negative impact on the infant’s personality and behavioral development, thus exerting long-term adverse effects on the infant’s future learning and life (21, 22). PPD can lead to significant impairments in both maternal functioning and mother-infant attachment, and these impairments can have lasting effects on the emotional and cognitive development of children (23, 24). This can affect the infants’ future emotional development and the establishment of social and interpersonal relationships (25). PPD has negative impacts on the whole family (26). PPD weakens the emotional connection between mother and baby, causes communication barriers between couples, exacerbates family role conflicts, and forms a negative interactive cycle, comprehensively disrupting the balance of family relationships (27). PPD causes emotional distress, adversely affects infant development and child adjustment, disruptions in family relationships, and financial burden (28, 29). Mothers with PPD may be unable to participate in work or social activities normally, damaging the family’s social relationships and affecting the family’s social status and interpersonal relationships (30, 31). The treatment and rehabilitation of PPD require certain tangible costs, such as therapy or medications, which can increase the family’s financial burden (32).

Early detection of symptoms and timely initiation of treatment can significantly reduce adverse outcomes (33, 34). In order to accurately predict PPD, many methods have been proposed (35, 36). Su et al. (37) used a logistic regression model to predict and explore the influencing factors of PPD in Chinese women and to construct a prediction model. Liu et al. (38) used 6 machine learning models to construct a PPD prediction model for cesarean section mothers, but this study only examined PPD in cesarean section mothers. Lin et al. (39) used multifactorial logistic regression analysis to identify independent factors affecting postpartum anxiety in early-onset preeclampsia and constructed a predictive model, with the model’s sensitivity, specificity, and accuracy being 81.82, 84.48, and 83.75%, respectively. However, this study only examined postpartum anxiety in women with preeclampsia. Chen and Shi (40) proposed a novel PPD prediction model based on the logistic regression model, which can effectively predict the risk of PPD in older adult pregnant women. However, this model only studies the PPD risk in older adult pregnant women, and its generalizability and interpretability are low. Lilhore et al. (41) proposed a novel PPD prediction framework that combines bidirectional long short-term memory and convolutional neural network-based transfer learning. However, the interpretability of this framework is not strong. Perry et al. (42) studied the relationship between maternal bipolar disorder and PPD. However, the small sample size limited the reliability of their findings. Matsumura et al. (43) used decision trees to predict chronic PPD in the Japanese context. Chen et al. (44) used a logistic regression model to study the prediction model of PPD in Chinese women. However, the machine learning models used in this study were too singular and did not explore the predictive effects of more machine learning models on PPD.

Although Support Vector Machine (SVM) can construct the optimal hyperplane to achieve high-precision disease prediction, when dealing with complex high-dimensional PPD related features, the computational complexity of SVM is too high, which can cause problems such as slow response time and difficulty in promoting SVM technology in practical clinical applications (45). K-Nearest Neighbors (KNN) relies on distance measures between data to predict PPD. However, KNN is sensitive to noise and outliers in the dataset, and is easily affected by local features of the data, leading to biased disease predictions (46). Decision Tree (DT) recursively divides the dataset into tree structures for PPD prediction, but DT is sensitive to changes in training data and prone to overfitting. When processing complex high-dimensional PPD datasets, the generated tree structures are too complex, resulting in insufficient generalization ability (47). Random Forest (RF) improves stability by integrating multiple DTs and can to some extent handle complex high-dimensional PPD datasets. However, the interpretability of RF is insufficient, making it difficult to clearly explain the specific contribution of each feature to the target feature to medical personnel, which is not conducive to the application and promotion of RF in clinical decision-making (48). Naive Bayes Classifier (NBC) is based on the assumption of independent features for PPD prediction, while the influencing factors of PPD are often interrelated, which is inconsistent with the application assumption of NBC and reduces the prediction accuracy and generalization ability of NBC (49). Logistic Regression (LR) is a linear model that requires high linear assumptions for the dataset, making it difficult to accurately obtain the complex nonlinear relationships between the influencing factors of PPD, which leads to inaccurate parameter estimation in LR and affects the prediction performance of PPD (27). Linear Discriminant Analysis (LDA) assumes that the data follows Gaussian distribution, which is often difficult to meet in actual PPD datasets, resulting in LDA being unable to accurately estimate the parameters of PPD. The accuracy of predicting PPD is not high (50). AdaBoost classifier can combine multiple weak classifiers to improve prediction performance, but the training process of AdaBoost overly relies on the selection of initial classifiers and is sensitive to noise and outliers in the training data, resulting in poor generalization ability in AdaBoost (51). Convolutional neural network (CNN) has high performance in predicting grid structured data, but PPD datasets come from a wide range of sources, and PPD data is generally not grid structured, making it difficult for CNN to fully explore the important information related to PPD dataset. Long Short-Term Memory (LSTM) has high performance in processing time series information, but PPD datasets are often non time series data, which limits the accuracy of LSTM in predicting PPD (52).

The combination of FCNN and DNN is of great necessity and rationality in predicting PPD. Due to the shortcomings of existing PPD prediction models in predicting PPD, such as low accuracy, poor generalization ability, and weak interpretability, accurate prediction of PPD faces many challenges. FCNN has a simple and direct structure, and the connection pattern between neurons in FCNN enables it to clearly reflect the relationship between features and target feature. FCNN has high interpretability, which helps medical personnel understand the disease prediction process in clinical practice. However, having too many parameters during the training process of FCNN can lead to high computational complexity and overfitting, resulting in poor generalization ability. Moreover, the Dropout mechanism in DNN randomly discards some neurons during the training process to effectively reduce the risk of overfitting and improve the generalization ability of DNN. During the training process, DNN needs to constantly reselect the discarded neurons, which reduces the interpretability of DNN.

Based on these considerations, this paper proposes a novel ensemble algorithm, which integrates FCNN and DNN. The use of Dropout mechanism reduces the probability of overfitting in the proposed model, thereby improving the generalization ability of the proposed model. FCNN enhances the interpretability and prediction accuracy of the proposed model. The proposed model is an ensemble algorithm that exhibits lower sensitivity to noise and outlier data compared to FCNN and DNN, demonstrating stronger stability. By optimizing weight allocation between FCNN and DNN, the model efficiently captures feature correlations within PPD datasets, achieving accurate and stable prediction results. This approach provides a more effective solution for PPD prediction tasks.

The main contributions of the proposed model are as follows:

- We integrate FCNN and DNN to propose a novel PPD integrated classifier.
- We integrate two weak neural network classifiers in the proposed model, effectively improving its PPD prediction accuracy.
- We utilize the Dropout mechanism in the proposed model to enhance its generalization ability.
- We use the PPD dataset from the KAGGLE platform to validate the performance of the proposed model in predicting PPD, demonstrating its representativeness and applicability.
- We demonstrate that, compared to various machine learning classifiers such as SVM, KNN, DT, RF, NBC, LR, LDA, AdaBoost, CNN and LSTM Classifier, the proposed model has higher accuracy and stability in predicting PPD.

The proposed model can have a significant impact on future medical practices, as neural network models have long demonstrated important potential in real-world clinical environments. For example, Kasmaee et al. (53) have integrated ensemble learning and reinforcement learning for effective diagnosis of myocarditis from CMR images, which addresses the main technical challenges of inherent data imbalance in cardiac magnetic resonance imaging datasets. Vyas and Khadatkar (54) have combined deep learning models with machine learning classifiers for diagnosing pneumonia can effectively improve the accuracy and efficiency of pneumonia diagnosis. Venkatesh et al. (55) have integrated one-dimensional CNN and bidirectional long short-term memory model, which have been used to diagnose atrial arrhythmia. The reliability of the model in accurately diagnosing atrial arrhythmia has been verified in real-time clinical applications. Sarayar et al. (56) has proven that CNNs have high potential in the diagnosis and classification of infectious keratitis. The proposed model is an ensemble model of two classic neural networks, which means that the proposed model integrates the advantages of FCNN and DNN. This means that the proposed model has high potential in solving problems in real-world clinical environments.

## 2 Method and dataset

### 2.1 Dataset

The dataset used in this paper comes from the KAGGLE platform and includes 1,503 records from a hospital, which were collected through questionnaires distributed to participants via Google Forms. This dataset has 15 features, and based on their relevance to the research objectives of this paper, 10 features were selected for study. 9 features are used as independent variables, and the target feature is “Feeling anxious. “. An interesting distinction that makes PPD unique from other depressive disorders is that it is marked by a prominent anxiety component. 66% of depressed mothers have a co-morbid anxiety disorder (57). Anxiety has been shown to be closely related to PPD (58). This attribute is selected for its potential utility as a predictive marker for PPD. Table 1 indicates the attributes and the relevant description during data collection of the dataset.

**Table 1:** Detailed description of this dataset.

No	Attribute	Description	Representation	Data encoding relationship
1	Age	Postpartum woman’s age after giving birth	25–30, 30–35, 35–40, 40–45, 45–50	1 = 25–30;
				2 = 30–35;
				3 = 35–40;
				4 = 40–45;
				5 = 45–50.
2	Feeling sad or Tearful	Does postpartum woman feel sad or tearful after giving birth?	Yes, no, sometimes	1 = yes;
				2 = sometimes;
				3 = no.
3	Irritable towards baby and partner	Is postpartum woman angry with baby or partner after giving birth?	Yes, no, sometimes	1 = yes;
				2 = sometimes;
				3 = no.
4	Trouble sleeping at night	Does postpartum woman feel sleep disorder after giving birth?	Yes, no, two or more days a week	1 = yes;
				2 = two or more days a week;
				3 = no.
5	Problems concentrating or making decision	Does postpartum woman have difficulty concentrating or making decisions after giving birth?	Yes, no, often	1 = yes;
				2 = often;
				3 = no.
6	Overeating or loss of appetite	Does postpartum woman have overeating or loss of appetite after giving birth?	Yes, no, not at all	1 = yes;
				2 = not at all;
				3 = no.
7	Suicide attempt	Did postpartum woman commit suicide after giving birth?	Yes, no, not interested to say	1 = yes;
				2 = not interested to say;
				3 = no.
8	Feeling of guilt	Does postpartum woman feel guilty after giving birth?	Yes, no, maybe	1 = yes;
				2 = maybe;
				3 = no.
9	Problems of bonding with baby	Does postpartum woman feel difficult to establish intimate relationship with her baby after giving birth?	Yes, no, sometimes	1 = yes;
				2 = sometimes;
				3 = no.
10	Feeling anxious	Does postpartum woman feel anxious after giving birth?	Yes, no	1 = yes;
				2 = no.

**Figure 1:**
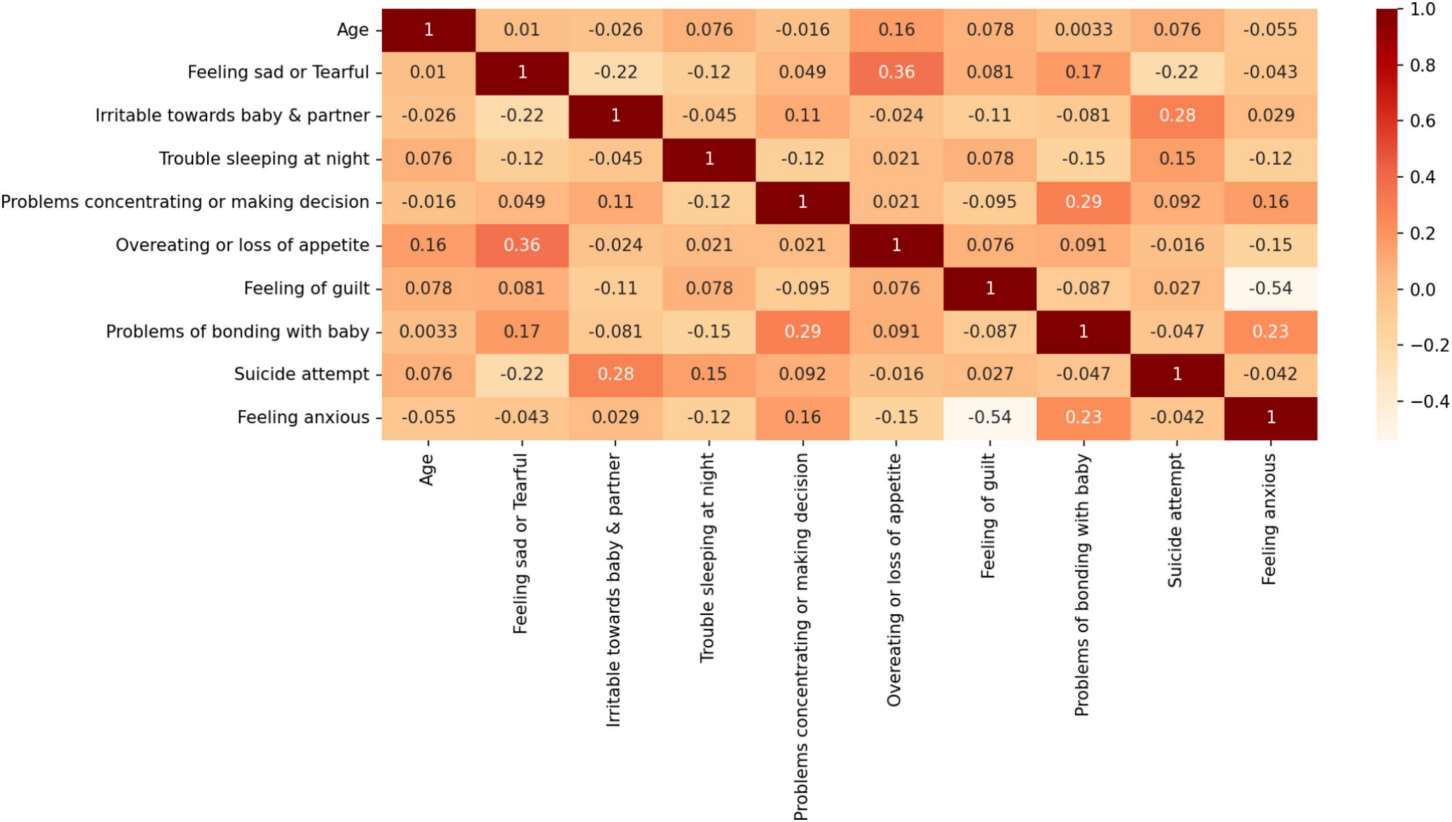
Pearson correlation coefficient diagram of the PPD dataset.

Table 1 lists the detailed information of the PPD dataset used in this paper and the corresponding encoding relationships. The target features in Table 1 are divided into two classes. One class is mothers with PPD, and the other is mothers without PPD. The number of mothers with PPD and the number of mothers without PPD account for 65 and 35% of the total number of cases in the dataset, respectively. During pregnancy and after childbirth, there are significant fluctuations in women’s hormones. During pregnancy, estrogen and progesterone are at high levels, but they drop rapidly after childbirth. This disrupts the normal functions of neurotransmitters in the brain, especially serotonin which affects mood regulation. Therefore, many women are more prone to anxiety symptoms. When becoming a mother for the first time, there is a huge psychological transformation. New mothers have to face many new responsibilities, worry about the health of their babies, and their self-identities also change. Worries about whether they can fulfill the role of a mother and lack of confidence in taking care of a newborn may all lead to an increase in anxiety. Therefore, it is somewhat reasonable that the number of mothers with PPD is greater than that of mothers without PPD. This further emphasizes the necessity of early identification and intervention for PPD. The Pearson correlation coefficient graph of the dataset in this paper is shown in Figure 1.

As depicted in Figure 1, the Pearson correlation analysis reveals the strongest pairwise association between the “overeating or loss of appetite” and “feeling sad or Tearful” features (r = 0.36). This observation suggests two critical implications for our analysis of PPD dataset: First, the relatively modest correlation magnitudes across all feature pairs indicate weak evidence for direct bivariate causality between individual symptom manifestations. Second, these findings reinforce the theoretical premise that PPD symptomatology likely emerges from multifactorial interactions rather than simple linear relationships between isolated symptoms. To account for this clinical complexity, we retained all nine features in our predictive modeling framework, hypothesizing that their multivariate integration through machine learning algorithms would yield greater predictive validity than any individual symptom measure alone.

### 2.2 The proposed model

#### 2.2.1 BP neural network

**Figure 2:**
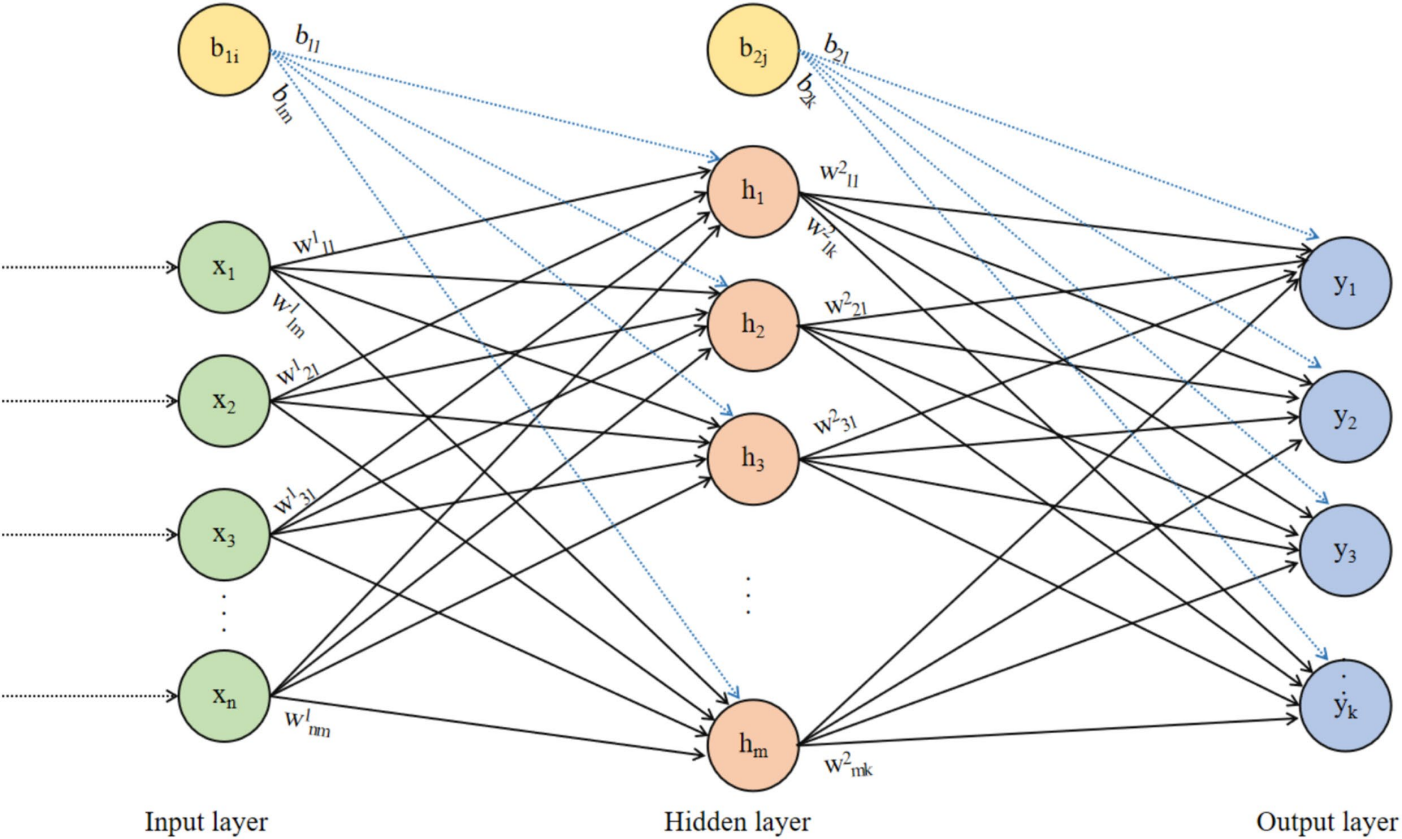
Structure diagram of the BP neural network.

The Back Propagation (BP) neural network was proposed by Rumelhart and McClelland in 1986 (59). It is a multilayer feedforward neural network trained using the error backpropagation algorithm and is currently the most widely used type of neural network. The BP neural network is a widely used feedforward neural network, suitable for various complex nonlinear problems. The structure diagram of the BP neural network is shown in Figure 2.

In Figure 2, the data transmission from the input layer to the hidden layer is as shown in Equation 1:


(1) $$h_{j}=f\left(\overset{n}{\underset{i=1}{\sum }}w_{ji}^{1}x_{i}+b_{1j}\right)$$

where, $x_{i}$ is the i-th neuron of the input layer, $w_{ji}^{1}$ is the weight from the i-th neuron of the input layer to the j-th neuron of the hidden layer, $b_{1j}$ is the bias of the j-th neuron of the hidden layer, and $f\left(\cdot \right)$ is the activation function of the hidden layer. The data transfer from the hidden layer to the output layer is as shown in Equation 2:


(2) $$y_{l}=f\left(\overset{m}{\underset{j=1}{\sum }}w_{lj}^{2}h_{j}+b_{2l}\right)$$

where, $w_{lj}^{2}$ is the weight from the j-th neuron in the hidden layer to the l-th neuron in the output layer, and $b_{2l}$ is the bias of the l-th neuron in the output layer. The prediction error of the BP neural network is as shown in Equation 3:


(3) $$E=\frac{1}{2}\overset{k}{\underset{l=1}{\sum }}{\left(y_{l}-d_{l}\right)}^{2}$$

where, $d_{l}$ is the actual output of the PPD dataset, and $y_{l}$ is the predicted output of the BP neural network.

The BP neural network uses backpropagation to train weights and biases, that is, by using the chain rule to propagate the output layer’s error back to the hidden layer and input layer, calculating the gradient for each weight and bias. The update for weight $w_{lj}^{g}$ is as follows in Equation 4:


(4) $$w_{ij}^{g}=w_{ij}^{g}-\eta \frac{\partial E}{\partial w_{ij}^{g}}$$

where, $w_{ij}^{g}$ is the weight from the j-th neuron of the g-th layer to the i-th neuron of the g + 1 layer, η is the learning rate, and $\frac{\partial E}{\partial w_{ij}^{g}}$ is the gradient of the prediction error with respect to the weight. The BP neural network in Figure 2 is an FCNN, where each neuron in each layer is connected to all neurons in the previous layer, making it easy to implement. Connections are established between adjacent layer neurons in FCNNs because FCNNs possess strong function approximation capabilities, allowing them to represent complex nonlinear mapping relationships. This enables FCNNs to handle various complex machine learning tasks, such as regression, classification, and clustering.

#### 2.2.2 The Dropout mechanism in neural networks

Each neuron in every layer of an FCNN is connected to all neurons in the previous and subsequent layers, which results in an excessive number of weights and bias parameters for the FCNN. This is especially true when dealing with high-dimensional input data, leading to high computational complexity for the FCNN. The large number of parameters that need to be trained makes the FCNN prone to overfitting.

**Figure 3:**
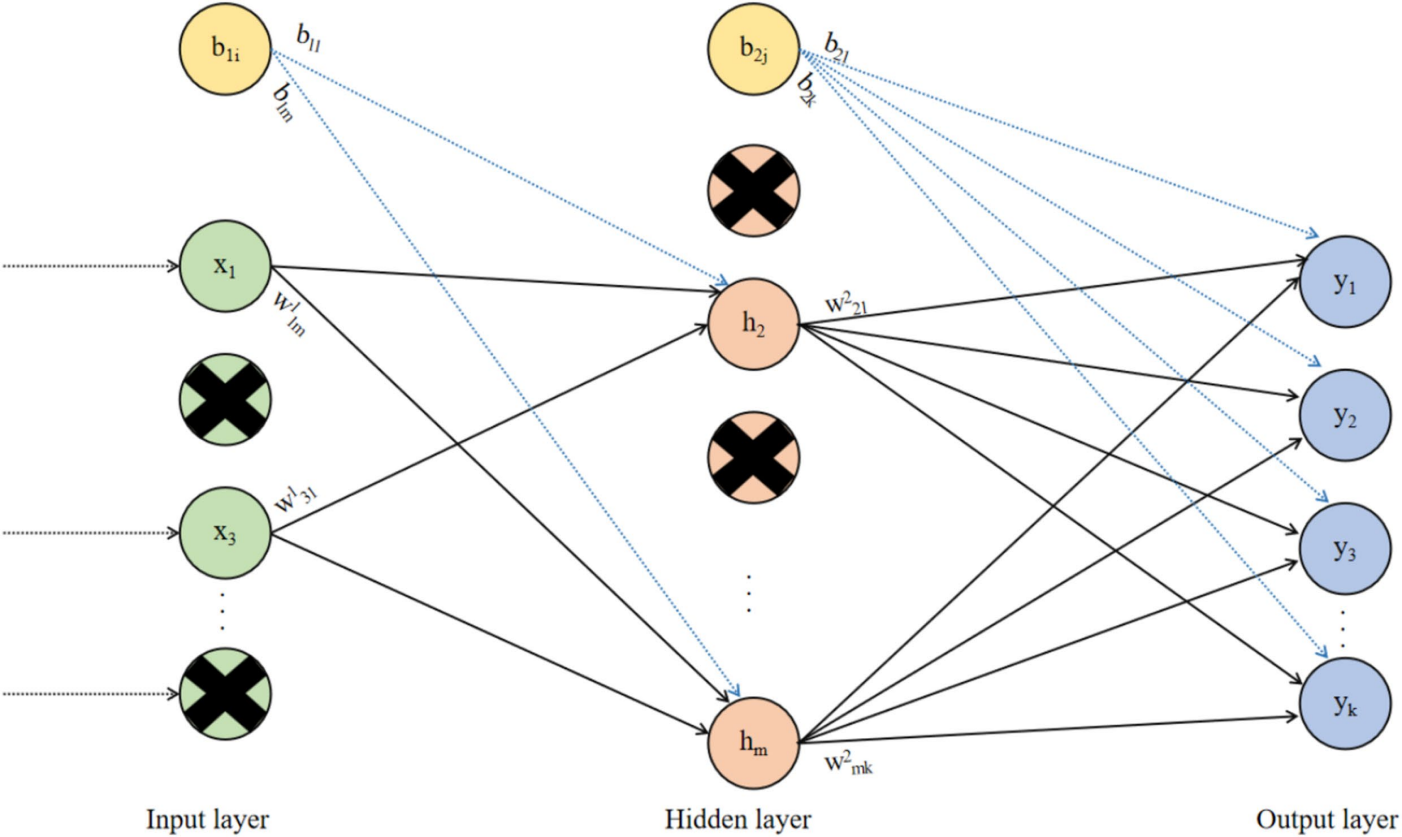
Structure diagram of a neural network with Dropout.

Based on the issues of excessive parameters and overfitting in FCNNs, the Dropout mechanism was proposed (60). The principle of Dropout is to randomly select a portion of neurons during the training process of the neural network and set their outputs to zero. This means that the structure of the neural network changes with each forward propagation, effectively reducing the overfitting phenomenon in the neural network. At the same time, because the Dropout mechanism randomly drops some neurons in the neural network, the number of weights and biases that the neural network needs to train is also reduced, thereby lowering the computational complexity of the neural network. The neural network based on the Dropout mechanism is shown in Figure 3.

In Figure 3, Dropout is a commonly used method to prevent neural networks from overfitting. By randomly removing some neurons during the training process of the neural network, it ensures that the network does not become overly dependent on the connections between certain neurons, thereby improving the network’s generalization ability and robustness, and enabling the neural network to perform excellently on unseen data.

#### 2.2.3 The proposed model

Each neuron in every layer in FCNN is connected to all neurons in the previous layer, which results in a lot of parameters such as weights and biases. When the input data has a high dimensionality, the computational complexity of the FCNN will become very high. FCNN requires training a large number of parameters, which is prone to overfitting. FCNNs can be affected by redundancy and detailed data in the dataset easily, which leads to poor generalization ability.

**Figure 4:**
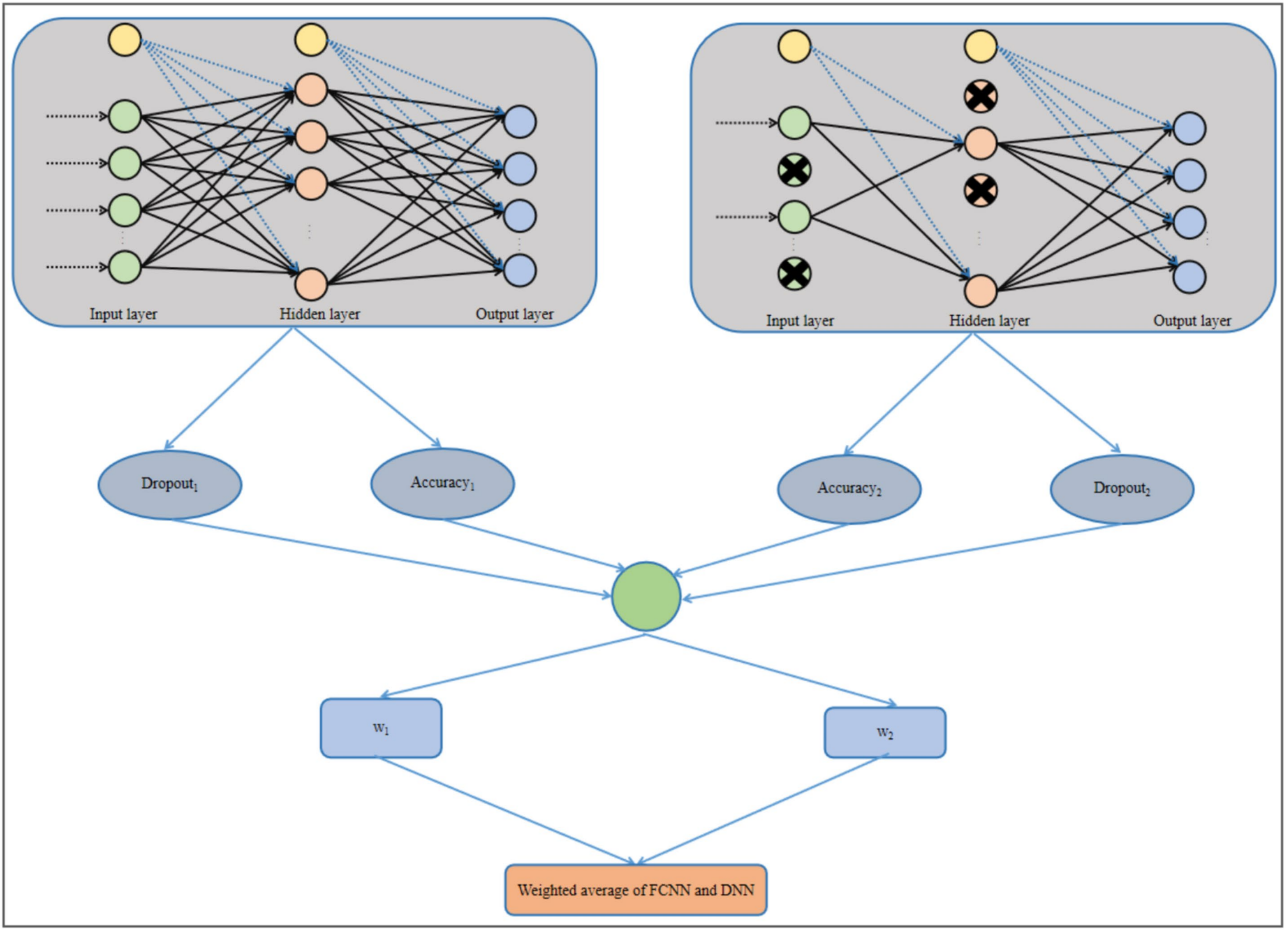
Structural diagram of the proposed model.

DNN needs to reselect which neurons to drop during each training iteration of the DNN, which will increase the training time of DNN. Different datasets require different Dropout rates for the DNN. If the Dropout rate is too high, it can cause the DNN to become unstable. If the Dropout rate is too low, it can lead the DNN to overfitting. If the chosen Dropout is not appropriate, it can lead to significant performance fluctuations during the training process of the DNN. Since the Dropout mechanism randomly drops neurons in the DNN, it reduces the interpretability of the DNN. Based on the respective problems of FCNN and DNN, this paper proposes a novel ensemble algorithm to combine the advantages of both and overcome the shortcomings of both. The structure of the proposed model is shown in Figure 4.

In Figure 4, a novel ensemble algorithm is proposed, which is a weighted average of FCNN and DNN. The PPD dataset has been divided into training and testing sets under the split ratio. The training set has been used to train the FCNN and DNN. After training, the accuracy of the FCNN and the DNN on the training set can be obtained, denoted as Accuracy_1_ and Accuracy_2_, respectively. Based on Accuracy_1_ and Accuracy_2_, the weights w_1_ and w_2_ for FCNN and DNN in the proposed model can be obtained, respectively, as shown in Equations 5, 6:


(5) $$w_{1}=\frac{\frac{\mathrm{Accurac}y_{1}}{\mathrm{Accurac}y_{1}+\mathrm{Accurac}y_{2}}+\frac{\mathrm{Dropou}t_{1}}{\mathrm{Dropou}t_{1}+\mathrm{Dropou}t_{2}}}{2}$$

(6) $$w_{2}=\frac{\frac{\mathrm{Accurac}y_{2}}{\mathrm{Accurac}y_{1}+\mathrm{Accurac}y_{2}}+\frac{\mathrm{Dropou}t_{2}}{\mathrm{Dropou}t_{1}+\mathrm{Dropou}t_{2}}}{2}$$

where, Dropout_1_ and Dropout_2_ are the Dropouts of FCNN and DNN on the training set, respectively. w_1_ and w_2_ are based on Accuracy_1_ and Accuracy_2_, as well as Dropout_1_ and Dropout_2_. For neural network with higher accuracy, the proportion it occupies in the proposed model is higher, which helps improve the prediction accuracy of the proposed model. For neural network with larger Dropout, the proportion it occupies in the proposed model is greater, which helps to enhance the algorithm’s generalization ability.

The proposed model is based on FCNN and DNN, which integrates the advantages of FCNN and DNN. The Dropout mechanism can reduce the overfitting risk of FCNN, make the proposed model more generalizable. Using the interpretability of FCNN ensures the interpretability of the proposed model. The training time of FCNN is less than that of DNN, and the computational complexity of DNN is lower than that of FCNN.

FCNN has a certain interpretability. In FCNN, the neurons in each layer are connected to all the neurons in the previous and next layers, which makes the structure of FCNN relatively simple and direct, making it easy to understand the relationships between data features in FCNN. The proposed model inherits the interpretability of FCNN. DNN uses Dropout mechanism to randomly drop neurons, which makes the network structure of DNN constantly change during training, and makes it difficult to confirm the specific role of each neuron and the connections between neurons in the final decision. It reduces the interpretability of DNN. However, although the proposed model inherits FCNN and DNN, it does not directly rely on the Dropout mechanism to prevent overfitting. By allocating weights reasonably, the proposed model’s structure is more stable compared to DNN, thereby greatly controlling the negative impact of Dropout on the interpretability of the proposed model. The proposed model determines the weights of FCNN and DNN in the proposed model based on their accuracy (Accuracy_1_ and Accuracy_2_) on the training set, as well as their Dropout values (Dropout_1_ and Dropout_2_). This weight allocation method is logical and interpretable. Neural network with higher accuracy on the training set has a larger proportion in the proposed model, because neural network with higher accuracy performs better in establishing connections between features and target features, and contributes more to the final prediction results. Neural network with larger Dropout value has a higher proportion in the proposed model because larger Dropout value helps enhance the model’s generalization ability. The proposed model combines the advantages of FCNN and DNN, which not only improves the prediction accuracy and generalization ability of the proposed model, but also has high interpretability.

The proposed model combines the advantages of FCNN and DNN. The Dropout mechanism in DNN reduces the risk of overfitting in the proposed model, enabling it to better adapt to different types of clinical data and avoid overfitting due to the specificity of the data during training. This improves the generalization ability of the proposed model to different types of data in actual clinical scenarios, thereby more accurately predicting PPD.

## 3 Results and discussions

### 3.1 Simulation experiment setup

The experiments in this paper were conducted on a server equipped with an Intel(R) Core(TM) i5-10210U CPU @ 1.60GHz (boosted to 2.11 GHz), using Python 3.13.1 as the programming language and PyCharm 2024. 1.7 as the programming software. The experimental data is sourced from the PPD dataset on the Kaggle platform (61) (link: https://www.Kaggle.com/code/xuexue12345/postpartum-depression-classification/edit), which details information about pregnant women and postpartum depression. Using SVM, KNN, DT, RF, NBC, LR, LDA, AdaBoost, CNN and LSTM Classifier, a performance comparison of the PPD dataset is conducted. The medical classification evaluation metrics used in this paper are accuracy, precision, recall, F1-score, MCC, specificity, NPV, FPR, and FNR.

### 3.2 The proposed model and performance analysis of classical machine learning classifiers

**Figure 5:**
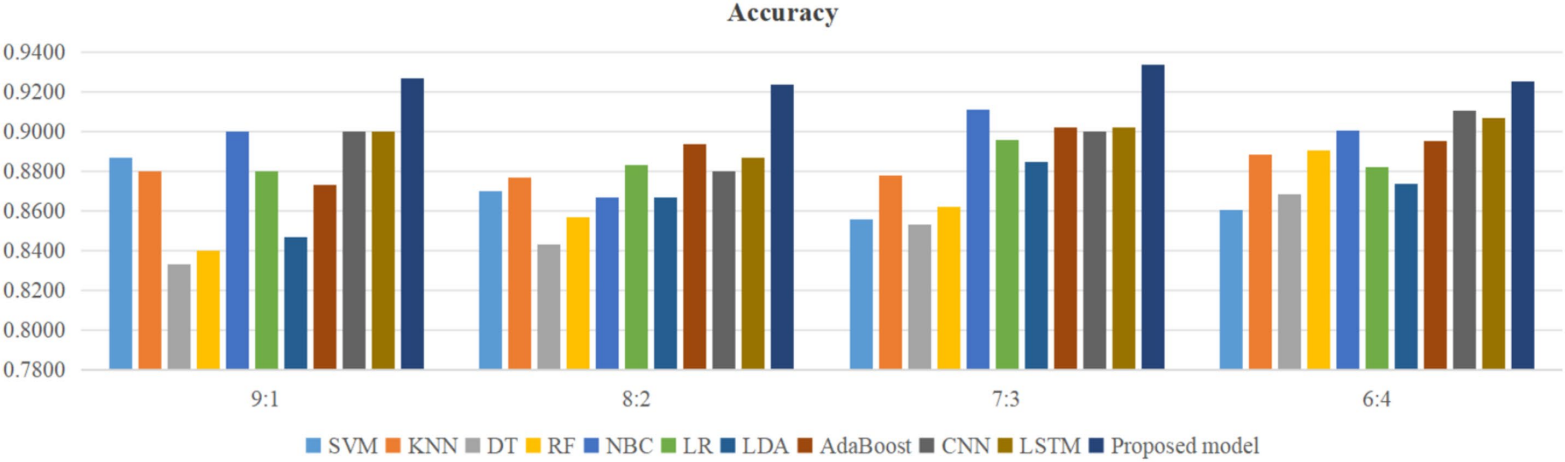
Comparison of accuracy between the proposed model and classical models.

**Figure 6:**
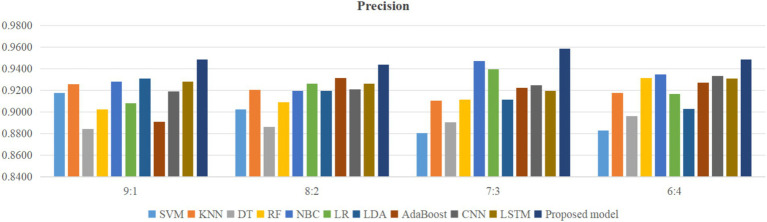
Comparison of precision between the proposed model and classical models.

**Figure 7:**
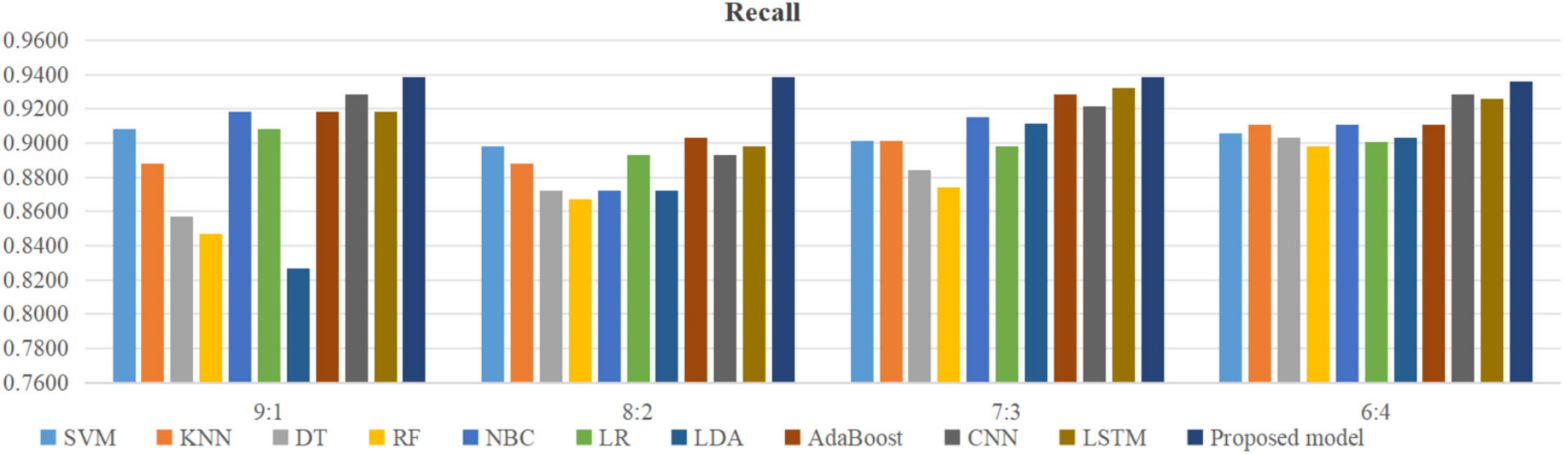
Comparison of recall between the proposed model and classical models.

**Figure 8:**
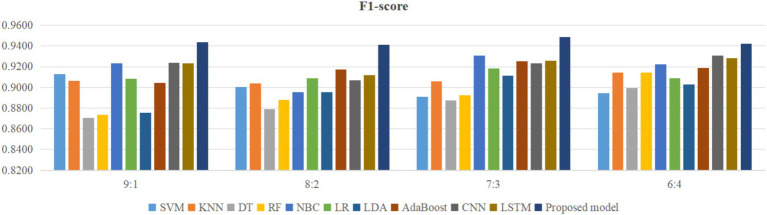
Comparison of F1-score between the proposed model and classical models.

Using four different dataset split ratios to divide the PPD dataset into training and testing sets, with split ratios of 9:1, 8:2, 7:3, and 6:4, respectively. Using the training set to train the proposed model and 10 classic machine learning classifiers, and using the test set to test the trained classifiers, the test results are shown in Figures 5–8.

From Figure 5, it can be seen that when the split ratios of the dataset in this paper are 9:1, 8:2, 7:3, and 6:4, compared to the other 10 classical classifiers, the accuracy of the proposed model is the highest. From Figure 6, it can be seen that when the split ratios of the dataset in this paper are 9:1, 8:2, 7:3, and 6:4, the proposed model has the highest precision compared to the other 10 classical classifiers. From Figure 7, it can be seen that when the split ratios of the dataset in this paper are 9:1, 8:2, 7:3, and 6:4, compared to the other 10 classical classifiers, the proposed model has the highest recall. From Figure 8, it can be seen that when the split ratios of the dataset in this paper are 9:1, 8:2, 7:3, and 6:4, compared to the other 10 classic classifiers, the F1-score of the proposed model is the highest. This indicates that the proposed model has the best PPD prediction performance compared to the other 10 classical classifiers under 4 split ratios. Meanwhile, because the proposed model achieved better predictive performance than the other 10 classifiers at 4 different split ratios, it indicates that the proposed model has higher stability in predicting PPD compared to the other 10 classifiers.

From Figure 5, it can be seen that the accuracy of the proposed model in this dataset is higher when the split ratio is 7:3 compared to the proposed model when the split ratios are 9:1, 8:2, and 6:4. From Figure 6, it can be seen that the proposed model has a higher precision in the split ratio of 7:3 compared to the proposed model with split ratios of 9:1, 8:2, and 6:4 in this PPD dataset. From Figure 7, it can be seen that the recall of the proposed model is equally large when the split ratio is 8:2 and 7:3 in this PPD dataset, and is greater than the recall of the proposed model when the split ratio is 9:1 and 6:4 in this PPD dataset. From Figure 8, it can be seen that the F1-score of the proposed model in the dataset with a split ratio of 7:3 is higher than that in the dataset with split ratios of 9:1, 8:2, and 6:4. In short, compared to split ratios of 9:1, 8:2, and 6:4, when the split ratio is 7:3, the proposed model has the highest accuracy, precision, and F1-score, So the split ratio of the PPD dataset used in this paper is 7:3. When the PPD split ratio is 7:3, the performance comparison of the proposed model with 10 machine learning classifiers under 9 evaluation metrics is shown in Table 2.

**Table 2:** Performance comparison of PPD prediction for dataset split ratio 7:3.

Models	Accuracy	Precision	Recall	F1-score	MCC	Specify	NPV	FPR	FNR
SVM	0.856	0.880	0.901	0.891	0.678	0.769	0.805	0.231	0.099
KNN	0.878	0.911	0.901	0.906	0.731	0.833	0.818	0.167	0.099
DT	0.853	0.890	0.884	0.887	0.677	0.795	0.785	0.205	0.116
RF	0.862	0.911	0.874	0.892	0.702	0.840	0.780	0.160	0.126
NBC	0.911	0.947	0.915	0.931	0.808	0.904	0.849	0.096	0.085
LR	0.896	0.940	0.898	0.918	0.775	0.891	0.822	0.109	0.102
LDA	0.884	0.912	0.912	0.912	0.745	0.833	0.833	0.167	0.088
AdaBoost	0.902	0.922	0.929	0.925	0.784	0.853	0.864	0.147	0.071
CNN	0.900	0.925	0.922	0.923	0.780	0.859	0.854	0.141	0.078
LSTM	0.902	0.919	0.932	0.926	0.783	0.846	0.868	0.154	0.068
Proposed model	**0.933**	**0.958**	**0.939**	**0.948**	**0.855**	**0.923**	**0.889**	**0.077**	**0.061**

Under the evaluation of the same evaluation indicator, we mark the model with the best performance among multiple models as bold values.

**Figure 9:**
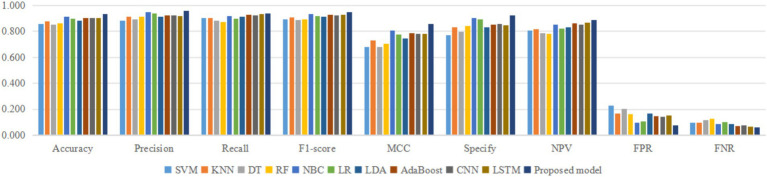
Comparison of 11 classifiers under 9 performance metrics.

As shown in Table 2, the accuracy, precision, recall, F1-score, MCC, specificity, NPV, FPR, and FNR of the proposed model are 0.933, 0.958, 0.939, 0.948, 0.855, 0.923, 0.889, 0.077, and 0.061, respectively. This indicates that the proposed model has higher values for accuracy, precision, recall, F1-score, MCC, specificity, and NPV, and lower FPR and FNR compared to the other 10 algorithms. This demonstrates that the proposed model exhibits more excellent prediction performance than the other 10 classic classifiers in terms of the 9 performance metrics. When the split ratio of the dataset in this paper is 7:3, the comparison of the 11 algorithms under the 9 performance metrics is shown in Figure 9.

Figure 9 intuitively shows that the proposed model has better indicators than the other 10 classifiers under each performance metric.

### 3.3 Statistical analysis

In order to eliminate the randomness in the predictions of these classifiers, this paper independently runs 11 classifiers, and each classifier needs to be run 30 times. Each time a classifier runs, the training set of the dataset in this paper is used to train the classifier, and then the test set of the dataset is used to test the trained classifier. The prediction accuracy of the classifier for the test set is recorded. Each classifier runs independently 30 times, that is, 30 prediction accuracies of the test set are obtained. The best, mean, median, worst, and standard deviation (STD) of these 30 prediction accuracies of the test set are recorded. And the Mann–Whitney U test is carried out between the proposed model and each of the 10 classifiers, respectively, to obtain the *p*-value as shown in Table 3.

**Table 3:** Comparative statistical analysis of PPD prediction.

Method	Worst	Mean	Median	Best	STD	*p*-value
SVM	0.702	0.856	0.855	0.897	0.095	<0.05
KNN	0.721	0.878	0.877	0.912	0.090	<0.05
DT	0.699	0.853	0.853	0.891	0.088	<0.05
RF	0.730	0.862	0.861	0.908	0.085	<0.05
NBC	0.796	0.911	0.910	0.943	0.075	<0.05
LR	0.805	0.896	0.895	0.937	0.070	<0.05
LDA	0.789	0.884	0.884	0.930	0.065	<0.05
AdaBoost	0.823	0.902	0.901	0.946	0.060	<0.05
CNN	0.828	0.900	0.895	0.932	0.060	<0.05
LSTM	0.821	0.902	0.895	0.935	0.059	<0.05
Proposed model	**0.846**	**0.933**	**0.933**	**0.950**	**0.055**	

Under the evaluation of the same evaluation indicator, we mark the model with the best performance among multiple models as bold values.

In Table 3, the proposed model obtained the worst, average, median, best, and standard deviations of 0.846, 0.933, 0.933, 0.950, and 0.055 in accuracy over 30 independent runs, respectively. Compared with the other 10 classifiers, the proposed model has higher mean and median, indicating that in most cases, the accuracy of the proposed model with the other 10 classes of PPD is higher. Compared with the other 10 classifiers, the proposed model has higher worst and best, indicating that even in the worst case, the lower limit of the proposed model’s ability to predict PPD is higher than the other 10 classifiers. At the same time, in the optimal situation, the proposed model’s performance upper limit for predicting PPD can also be higher than the other 10 classifiers. The proposed model, compared to the other 10 classifiers, has a lower STD, which indicates that the proposed model has more stable predictive performance in predicting PPD. The *p*-values of the Mann–Whitney U test for the proposed model and the other 10 classifiers are all less than 0.05, indicating a significant difference in accuracy between the proposed model and the other 10 classifiers in predicting PPD, excluding the influence of random errors. This result further confirms that the superiority of the proposed model is not accidental, but has a reproducible scientific basis.

### 3.4 Ablation experiment

The proposed model integrates FCNN and DNN, combining the advantages of both. This paper conducts ablation experiments on the proposed model as shown in Table 4.

**Table 4:** Ablation experiment of PPD prediction.

Metrics	FCNN	DNN	Proposed model
Accuracy	0.898	0.902	**0.933**
Precision	0.919	0.914	**0.958**
Recall	0.925	0.939	**0.939**
F1-score	0.922	0.926	**0.949**
MCC	0.774	0.782	**0.855**
Specify	0.846	0.833	**0.923**
NPV	0.857	0.878	**0.889**
FPR	0.154	0.167	**0.077**
FNR	0.075	0.061	**0.061**

Under the evaluation of the same evaluation indicator, we mark the model with the best performance among multiple models as bold values.

Table 4 shows that the proposed model exhibits significant advantages in the PPD prediction. As shown in Table 4, compared to FCNN and DNN, the proposed model achieved comprehensive optimization in key performance metrics: accuracy reached 0.933 (an increase of 3.4–3.9%), precision reached 0.958 (an increase of 4.2–4.8%), recall reached 0.939 (an increase of 0–1.5%), F1-score reached 0.949 (an increase of 2.5–2.9%), MCC reached 0.855 (an increase of 9.3–10.5%), Specify reached 0.923 (an increase of 9.1–10.8%), and NPV reached 0.889 (an increase of 1.3–3.7%). The FPR has been reduced to 0.077, effectively controlling the risk of false positives. Although the recall rate and FNR of the proposed model are on par with DNN, a recall of 0.939 and FNR of 0.061 are already impressive performance indicators. The experimental results confirmed that the proposed model can more comprehensively capture the complex features of PPD, providing reliable technical support for early clinical screening.

## 4 Conclusion

The proposed model integrates FCNN and DNN, which have strong anti-overfitting capabilities and interpretability. The proposed model has inherited the interpretability of FCNN, and utilized the Dropout mechanism of DNN to improve the generalization ability and anti overfitting capabilities of the proposed model. This experiment shows that compared to other classical classifiers, the proposed model has higher predictive performance on PPD prediction. This provides guidance and suggestions for the decision-making of medical participants related to PPD diagnosis.

In the future, the proposed model will be used to solve more practical clinical problems. The future work plan includes: (1) Further improve the proposed model by introducing adaptive Dropout to the proposed model, and compare the PPD prediction accuracy of the proposed model with and without variable Dropout values through experiments. (2) Use Google Forms to collect PPD related datasets, detailing questionnaire design, participant selection methods, ethical implications, potential biases, and hospital sources. (3) Collect more types of disease datasets, use the proposed model to predict diseases on these datasets, and further test the performance of the proposed model in solving practical clinical prediction problems. (4) Use the proposed model to achieve medical multimodal data fusion, that is, use FCNN and DNN to process one type of medical data separately, and fuse the processed data. (5) Establish a network PPD prediction platform, first embed the algorithm logic of the proposed model into the platform, and store data on various diseases on this platform. Then use the data in this platform to train the proposed model. Finally, patients can log in to the cloud platform in real time to input their own disease characteristics and use them to predict whether the user has related diseases.

## Data Availability

Publicly available datasets were analyzed in this study. This data can be found here: https://www.kaggle.com/datasets/parvezalmuqtadir2348/postpartum-depression.
